# Total kidney volume in autosomal dominant polycystic kidney disease: intraobserver and interobserver agreement of two methods with MRI

**DOI:** 10.55730/1300-0144.5820

**Published:** 2024-01-05

**Authors:** Elif GÜNDOĞDU, Çağatay CİHAN, Celal YAZICI

**Affiliations:** Department of Radiology, Faculty of Medicine, Eskişehir Osmangazi University, Eskişehir, Turkiye

**Keywords:** Autosomal dominant polycystic kidney disease, total kidney volume, magnetic resonance imaging, manual boundary tracing method, ellipsoid formula

## Abstract

**Background/aim:**

Total kidney volume (TKV) is a parameter used in both treatment decision and follow-up in autosomal dominant polycystic kidney disease (ADPKD) patients. The objective of this study was to evaluate intra- and interobserver agreement of the ellipsoid formula (EF) and manual boundary tracing method (MBTM) used in TKV measurement of ADPKD patients across different levels of experience radiologists. Additionally, the study aimed to evaluate the correlation between the EF and MBTM, which is considered the gold standard for TKV.

**Materials and methods:**

A retrospective evaluation was conducted on magnetic resonance imaging (MRI) data from 55 ADPKD patients who underwent abdominal MRI between January 2017 and November 2021 to evaluate TKV. TKV measurements were performed by three independent observers (observer 1, an abdominal imaging radiologist with 5 years of experience; observer 2, a fourth-year radiology resident; observer 3, a second-year radiology resident).

To assess intraobserver variability, all observers repeated the measurements at two-week intervals. The ICC was used to assess both intraobserver and interobserver variability. A comparison of the two methods was performed by linear regression for all three observers.

**Results:**

The ICC (95% CI) indicated excellent agreement between the observers for both methods (among all observers, p < 0.001). Furthermore, excellent intraobserver agreement was found between all observer measurements either EF or MBTM based on ICC (95% CI) (p < 0.001). The results of the linear regression analysis demonstrated high correlations between the two methods in all three observers (r = 0.992, p < 0.001 for the first observer; r = 0.975, p < 0.001 for the second observer; r = 0.989, p < 0.001 for the third observer).

**Conclusion:**

Both the EF and MBTM methods used for the measurement of TKV provided excellent intra- and interobserver reproducibility. The EF is as accurate and precise as the MBTM. It may therefore be preferred in radiology departments with heavy workload, as it is a reliable method for rapid and easy assessment, independent of experience.

## 1. Introduction

Autosomal dominant polycystic kidney disease (ADPKD) is a genetic and systemic disease characterized by multiple cysts developing in the kidneys and progressive loss of kidney functions with an increase in total kidney volume (TKV) [1,2]. Currently, there is no definitive treatment for this disease [3]. Some preventive measures, such as salt restriction, weight control, and increasing fluid intake, are the first steps in treatment. However, some antihypertensive agents, especially angiotensin converting enzyme inhibitors, and lipid-lowering agents are used in the treatment of the disease [4]. The vasopressin-2 receptor antagonist (tolvaptan), which is effective on the pathophysiological mechanism responsible for cyst formation, is one of the pharmacological agents that has been recently used [5]. The Tolvaptan Efficacy and Safety in Management of Autosomal Dominant Polycystic Kidney Disease and its Outcomes (TEMPO) 3:4 and 4:4 studies show that the use of tolvaptan slows renal disease progression in patients with advanced ADPKD, and there is a decrease in TKV in patients receiving tolvaptan after 3 years of follow-up [6,7]. In the TEMPO 3:4 study, it was stated that it would be appropriate for the patient group aged 18–55 years with an estimated glomerular filtration rate (eGFR) above 60 mL/min and a TKV above 750 mL to receive tolvaptan treatment [6]. TKV is used for both treatment decision and follow-up in ADPKD patients.

Magnetic resonance imaging (MRI) is accepted as the gold standard method for TKV measurement in the literature [8,9]. TKV volume can be calculated in two ways in MRI: ellipsoid formula (EF) and manual boundary tracing method (MBTM). EF is a generally accepted practical volume measurement method of spherical or oval shaped structures that is frequently used in daily radiology practice. MBTM is a standard volume measurement method that can be used to measure the volume of any shaped organ, but it requires a longer time [10]. Although MBTM for TKV is the gold standard technique, it is a time-consuming method and requires special software [10]. Due to these disadvantages, it is difficult to implement in practice. EF, on the other hand, is less time-consuming and does not require any special software. It is therefore a method preferred by radiologists in daily practice. Repeatability is one of the most important parameters that determine the reliability of different measurement methods. Therefore, the aim of this study was to investigate the intra- and interobserver agreement of the EF and MBTM used to measure TKV in patients with ADPKD by radiologists with different levels of experience. In addition, since MBTM is considered the gold standard for TKV, the correlation of EF with this method was also evaluated.

## 2. Materials and methods

The study was approved by the Ethics Committee of the Faculty of Medicine of Eskişehir Osmangazi University (Date: 22 December 2021 No: E-25403353-050.99-266323). The study was conducted in accordance with the principles of the Helsinki Declaration. All image data used in this study were obtained from routine imaging at our institution. Datasets were evaluated retrospectively. Therefore, approval and informed consent were not necessary and were waived by our local institutional review board.

### 2.1. Study participants

The MRI of ADPKD patients who underwent abdominal MRI for evaluation of TKV between January 2017 and November 2021 were retrospectively evaluated. Patients with MRI in which it was not possible to evaluate TKV due to motion artifacts (n = 2) or an inappropriate MRI (n = 3 not the whole kidney in the imaging area) were excluded from the study. The MRI scans of the remaining 55 patients were included in the study.

### 2.2. Image acquisition, analysis, and interpretation

All MRI scans were performed on a 3 T (General Electric) MRI scanner device using a 48-channel body coil. No contrast material was used in any of the patients. Axial plane T1-weighted gradient echo, T2-weighted single-shot fast spin-echo sequences in the axial, coronal and sagittal planes were obtained. The images were evaluated by radiologists using a dedicated workstation (Advantage WorkStation AW 4.7 software, GE Healthcare, WI, USA). Measurements were performed by three independent observers (observer 1, an abdominal imaging radiologist with 5 years of experience; observer 2, a fourth-year radiology resident; observer 3, a second-year radiology resident). Each observer conducted two measurements for each parameter, from which the average values were obtained. To assess intraobserver variability, observers repeated the measurements at two-week intervals. The volumes of the right and left kidneys were calculated separately, and then TKV was determined by summing them. A total of 110 kidneys in 55 patients were evaluated; all patients had two kidneys, and none had a solitary kidney). T2-weighted single-shot fast spin-echo sequences were used for all measurements.

**Figure 1:**
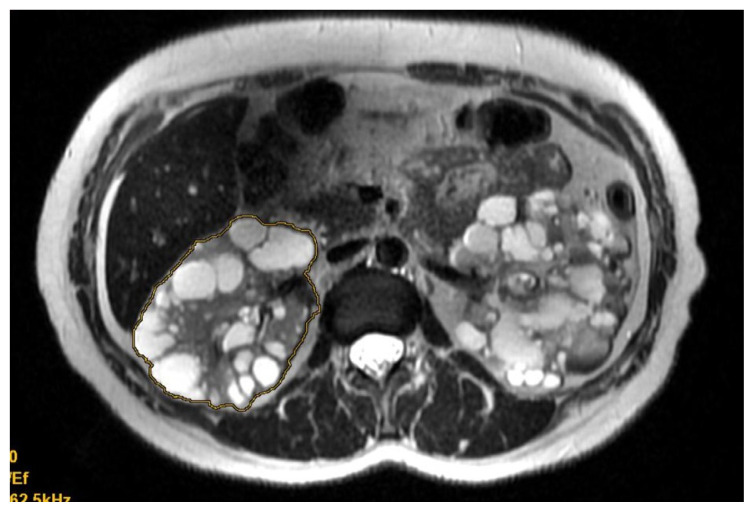
MBTM for TKV of ADPKD: kidney boundaries manually drawn on axial plane T2-weighted MRI.

For MBTM, both kidney boundaries were drawn manually on the axial plane of each slice (Figure 1). Kidney volumes were calculated from the set of contiguous images by summing the products of the area measurements within the kidney boundaries and slice thickness. Kidney volume was obtained automatically with software.

**Figure 2:**
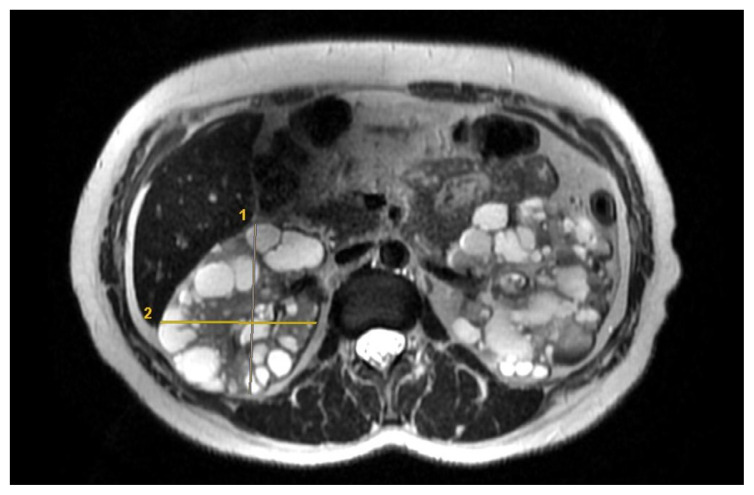
EF for TKV of ADPKD: The width from the axial plane image at maximum transversal diameter, and depth from the same image perpendicular to the width measurement.

The recommendation of the Mayo Clinic^1^ was used for the EF (π/6 × Lenght (Coronal Lenght +Sagittal Length)/2 × Depth × Width). Parameters are obtained from the 4 measurements using the axial, coronal, and sagittal planes. For each kidney, length was measured as the average maximal longitudinal diameter measured in the coronal and sagittal plane. Width was obtained from the transversal image at maximum transversal diameter, and depth was measured from the same image perpendicular to the width measurement (Figure 2).

### 2.3. Statistical analysis

SPSS software v. 22.0 (IBM Corporation, Armonk, NY, USA) was used for statistical analysis. Normality analysis was performed by the Shapiro-Wilk test. The mean, standard deviation (SD), minimum and maximum values were obtained as descriptive statistics of continuous data, and frequency (percentage) values for discrete data. The intraclass correlation coefficient (ICC) was used to assess intra- and interobserver variability. Based on the 95% confidence interval (CI) of the ICC estimate, values less than 0.5, ranging from 0.5 to 0.75, 0.75 to 0.9, and greater than 0.90 indicate poor, moderate, good, and excellent reliability, respectively. A comparison of two methods for TKV was performed by linear regression for all three observers.

## 3. Results

The study included 55 patients, of whom 26 (47.2%) were female and 29 (52.7%) were male. The mean age of the patients participating in the study was 47.36 ± 12.28 (25–80) years. Descriptive statistics of TKV calculated using the EF and MBTM and measured by the first, second, and third observers are presented in Table 1.

The ICC (95% CI) indicated excellent agreement between the observers for both methods (among all observers, p < 0.001). Furthermore, excellent intraobserver agreement was found between all observers’ measurements of either EF or MBTM on ICC (95% CI) (p < 0.001). Tables 2 and 3 show detailed information on intra- and interobservers agreement.

**Figure 3:**
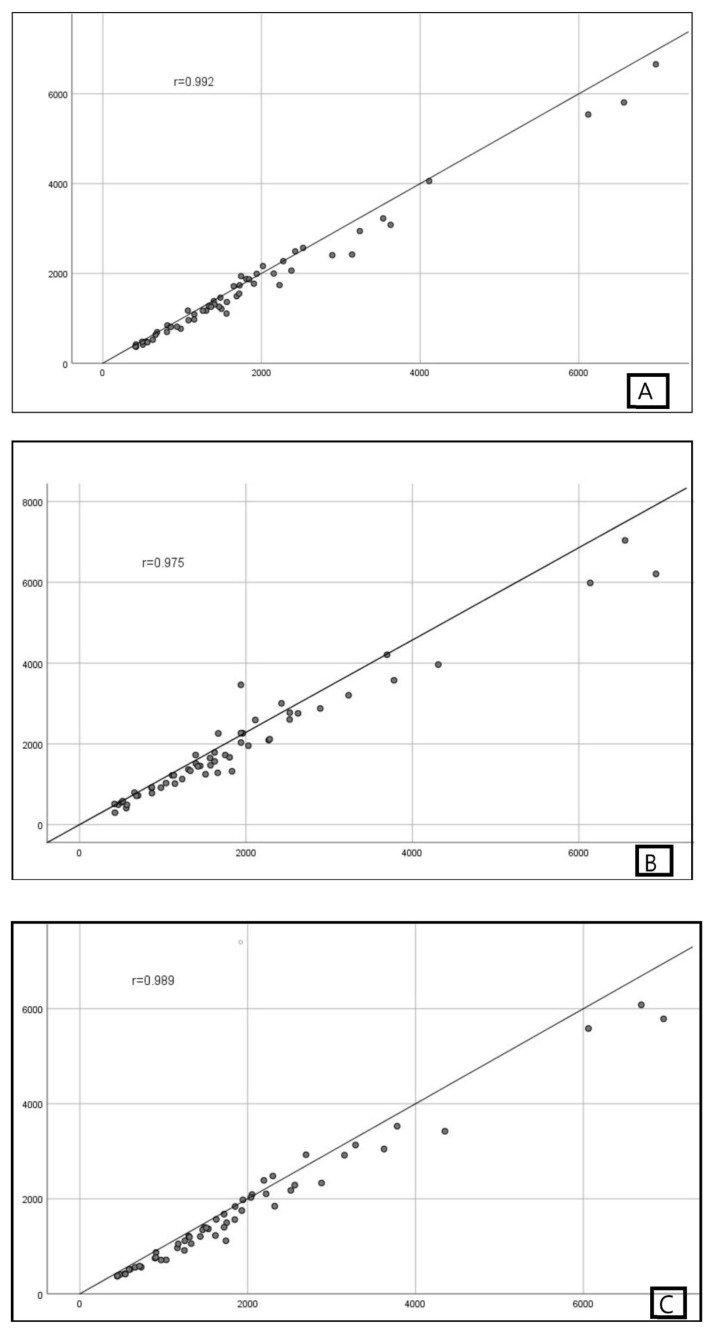
Linear regression analysis of measurement methods for all three observers A) first observer, B) second observer, and C) third observer.

Linear regression analysis was performed for all three observers to assess the correlation of measurement methods. High correlations were observed for two methods in all three observers (r = 0.992, p < 0.001 for the first observer; r = 0.975, p < 0.001 for the second observer; r = 0.989, p < 0.001 for the third observer) (Figure 3A, 3B, 3C).

## 4. Discussion

In this study, we evaluated the intra- and interobserver agreement levels and the correlation between the two methods (EF and MBTM) for determining TKV in ADPKD patients by radiologists with different levels of experience. We found that both the EF and MBTM had excellent intra- and interobserver agreement. The correlation of the EF with the MBTM, which is considered the gold standard for TKV, was also very high.

In the literature, there are some studies using different radiological methods to calculate TKV volume in ADPKD patients [9]. Ultrasonography (USG), despite its advantages such as being cheap, easily accessible, and not containing ionizing radiation, is not a precise and accurate method suitable this purpose [11,12]. Despite the advantage of short time of computed tomography (CT) application, its use in practice is limited (except in patients who cannot undergo MRI) due to ionizing radiation exposure, which poses a problem especially with repetitive examinations, and the difficulty in using iodinated contrast material in patients with impaired renal function [12]. MRI is the most appropriate imaging method used for this purpose because of its high soft tissue contrast resolution and the ability to easily identify renal borders and cysts without the need for contrast material. In the Consortium for Radiologic Imaging Studies of Polycystic Kidney Disease (CRISP) study, it was found that there were differences in TKV in measurements made with contrast and noncontrast T1-weighted images [13]. Today, T2-weighted sequences have replaced T1-weighted sequences due to the risk of nephrogenic systemic fibrosis of gadolinium-containing contrast agents and the rapid acquisition of T2-weighted sequences in parallel with recent technological developments. In our study, we also performed TKV measurements on T2-weighted sequences.

The gold standard method for TKV is MBTM performed on MR images [10]. In the literature, studies on this subject have shown that this method has high reproducibility rates. However, it is a time-consuming method and requires a specialized workstation [14]. Due to their heavy workload, radiologists need a less time-consuming and accurate method that can be applied in daily practice. For this purpose, studies have been conducted to evaluate whether the EF can be used due to the short evaluation time compared to the MBTM.

In their study, Higashihara et al. found that intra- and interobserver reliabilities for standard TKV and TKV calculated with EF were highly reliable [14]. Irazabal et al. maintain that TKV calculated with the EF is strongly correlated with TKV calculated by the stereological method (R^2^ = 0.979) [15]. In our study, we found a strongly correlation for all three observers, regardless of experience, as in this study (r = 0.992, p < 0.001 for the first observer; r = 0.975, p < 0.001 for the second observer; r = 0.989, p < 0.001 for the third observer). In addition to this study, we also found that the intra- and interobserver agreement of the EF was excellent and independent of experience. Cohen et al. found that while intraobserver agreement with the semiautomatic MR volumetric method was excellent, the interobserver agreement was quite good [16]. They suggested that the reason why the interobserver agreement is lower than the intraobserver agreement is that the readers have different experiences and that formal training at the workstation is insufficient. We found excellent intra- and interobserver agreements with both MBTM and EF, and we therefore think that this is independent of experience. Sharma et al., in their study with expert and beginner level observers, found high intraobserver variability in the beginner operator and reported that the measurements should be made by the expert operator [17]. Kidney volumes were performed on T1-weighted images in this study. Kidney cysts and their borders are more difficult to distinguish on T1-weighted images than on T2-weighted images. Therefore, fast T2-weighted sequences have been used for this purpose in recent studies. The high intraobserver variability of the beginner operator may be due to this. Also, the operators in this study are not radiologists. Nonradiologist operators may not be as familiar with MR images as radiologists. This may be another reason for the inconsistency with our study.

In recent years, there have been studies conducted with artificial intelligence (AI) applications for automatic kidney segmentation in ADPKD patients. Kline et al. found that the AI segmentation system they developed performed equally well with the readers [18]. Goel et al. found that the model-assisted segmentation they developed using the deep learning method required 51% less time than the manual contour determination method without model support [19]. These studies with AI are very promising for the future; however, full stomachs, full bladders, hemorrhagic renal cysts, and cysts located at the liver borders are still the cause of significant failure [19]. We think that the validity and widespread use of these studies, which are obtained through AI applications, will take time. It appears that radiologists will continue to spend time measuring volume in ADPKD patients in the near future, just as they do today. Although we did not record the evaluation times for the MBTM and EF, the average time for the MBTM in the literature is between 28 and 90 min [20]. On the other hand, 5–7 min are reported for the EF [21]. The MBTM requires 4–18 times more time than the EF. According to the results of our study, the EF is a time-efficient method that can be used safely by radiologists with different levels of experience. We can also speculate that the EF is more preferable among radiologists due to the increasing workload and the MBTM being the tedious contouring task. Of course, the most important issues are repeatability and accuracy. The result of our study may help radiologists in this preference.

The most important limitation of the study is its retrospective nature. Obtaining data from a single center is another limitation. In our study, all MRI examinations were conducted using a 3 T MRI device. Three-tesla scanners have a higher magnetic field strength and provide a higher signal-to-noise ratio, thus offering better image quality and cyst contrast [21]. To ensure the validity of our study results at 1.5 T, it may be necessary to support studies using MRI devices with this magnetic field strength.

In conclusion, both of methods (MBTM and EF) used in this study provided excellent intra- and interobserver reproducibility. EF is as accurate and precise as MBTM and it is a reliable method for rapid and easy assessment independent of experience. It may be preferred in radiology departments with heavy workload.

## Figures and Tables

**Table 1:** Descriptive statistics of TKV.

	Observer 1 Mean ± SD (cm^3^) Min-Max (cm^3^)	Observer 2 Mean ± SD (cm^3^) Min-Max (cm^3^)	Observer 3 Mean ± SD (cm^3^) Min-Max (cm^3^)
First measurement (EF)	1714.85 ± 1318.65 365–6658	1935.80 ± 1437.04 296–7039	1718.42 ± 1294.40 370–6082
Second measurement (EF)	1782.75 ± 1369.74 350–7073	2008.33 ± 1563.58 412–7677	1698.42 ± 1235.79 328–5680
First measurement (MBTM)	1855.96 ± 1431.10 410–6971	1886.89 ± 1425.64 419–6927	1927.85 ± 1434.78 446–6956
Second measurement (MBTM)	1845.53 ± 1410.50 196–6840	1911.44 ± 1451.55 412–6970	1980.47 ± 1469.95 438–7065

* TKV, total kidney volume; EF, ellipsoid formula; MBTM, manual boundary tracing method; SD, standard deviation; Min, minimum; Max, maximum.

**Table 2:** ICC Statistics for intraobserver agreement.

	ICC	95% confidence interval	p value
Observer 1 (EF)	0.98	0.97–0.99	0.0001
Observer 1 (MBTM)	0.99	0.99–0.99	0.0001
Observer 2 (EF)	0.97	0.96–0.98	0.0001
Observer 2 (MBTM)	0.99	0.98–0.99	0.0001
Observer 3 (EF)	0.99	0.98–0.99	0.0001
Observer 3 (MBTM)	0.99	0.99–0.99	0.0001

* ICC, intraclass correlation coefficient; EF, ellipsoid formula; MBTM, manual boundary tracing method.

**Table 3:** ICC Statistics for interobserver agreement.

	ICC	95% confidence interval	p value
Observer 1-2 (EF)	0.97	0.95–0.98	0.0001
Observer 1-2 (MBTM)	0.98	0.96–0.98	0.0001
Observer 1-3 (EF)	0.98	0.97–0.99	0.0001
Observer 1-3 (MBTM)	0.98	0.98–0.99	0.0001
Observer 2-3 (EF)	0.98	0.96–0.98	0.0001
Observer 2-3 (MBTM)	0.99	0.98–0.99	0.0001

* ICC, intraclass correlation coefficient; EF, ellipsoid formula; MBTM, manual boundary tracing method.

## Footnotes

1 Mayo Clinic (2013). Imaging classification of ADPKD: a simple model for selecting patients for clinical trials [online]. Website https://www.mayo.edu/research/documents/pkd-center-adpkd-classification/doc-20094754 [accessed 28 January 2023].
